# Recent Advances in Lignocellulose-Based Monomers and Their Polymerization

**DOI:** 10.3390/polym15040829

**Published:** 2023-02-07

**Authors:** Fuyun Pei, Lijuan Liu, Huie Zhu, Haixin Guo

**Affiliations:** 1 CECEP Techand Ecology & Environment Co., Ltd., Shenzhen 518004, China; 2 Graduate School of Engineering, Tohoku University, Sendai 980-8579, Japan; 3 Agro-Environmental Protection Institute, Ministry of Agriculture and Rural Affairs, Tianjin 300191, China

**Keywords:** cellulose, HMF, vanillin, bio-based polymers, biopolyester

## Abstract

Replacing fossil-based polymers with renewable bio-based polymers is one of the most promising ways to solve the environmental issues and climate change we human beings are facing. The production of new lignocellulose-based polymers involves five steps, including (1) fractionation of lignocellulose into cellulose, hemicellulose, and lignin; (2) depolymerization of the fractionated cellulose, hemicellulose, and lignin into carbohydrates and aromatic compounds; (3) catalytic or thermal conversion of the depolymerized carbohydrates and aromatic compounds to platform chemicals; (4) further conversion of the platform chemicals to the desired bio-based monomers; (5) polymerization of the above monomers to bio-based polymers by suitable polymerization methods. This review article will focus on the progress of bio-based monomers derived from lignocellulose, in particular the preparation of bio-based monomers from 5-hydroxymethylfurfural (5-HMF) and vanillin, and their polymerization methods. The latest research progress and application scenarios of related bio-based polymeric materials will be also discussed, as well as future trends in bio-based polymers.

## 1. Introduction

Since the industrial revolution, large-scale use of fossil resources has led to serious environmental problems such as the greenhouse effect, atmospheric pollution, soil, and marine pollution. Among these, cheap, lightweight, and versatile polymers are essential for all aspects of daily life, but most polymers are still highly dependent on chemicals derived from fossil fuels for their production [1]. Not only is the polymer industry facing a depletion of raw materials, but the production process also has an irreversible negative impact on the environment. For example, studies have shown that the equivalent CO_2_ emissions per kilogram of polypropylene and polyethylene produced are 1.34 and 1.48 kg, respectively [2]. In addition to this, only 14% of plastic packaging is collected for recycling after use and a significant amount escapes into the environment [3]. The resulting white pollution and greenhouse effect also pose an unprecedented challenge for industrial development and materials research and development. For this issue, mass production and applications of bio-based polymer materials through the efficient use of green and renewable plant resources are the most promising means for solving the problems [4]. The research and development of bio-based polymer materials have been well developed in recent years in terms of bio-based monomer preparation with high efficiency, novel polymerization methods of bio-based monomers, and the preparation of novel catalysts. Depending on the synthetic methods, bio-based polymers include two main categories: natural polymers and bio-based synthetic polymers from bio-based monomers [5]. Natural polymers are mainly represented by cellulose and lignin, etc., which are widely found in plants. For the latter category, the mainly used raw materials for bio-based synthetic polymers are lignocellulose, cellulose, hemicellulose, and lignin from biomass, which can be converted to polymerizable monomers through various chemical processes.

**Figure 1:**
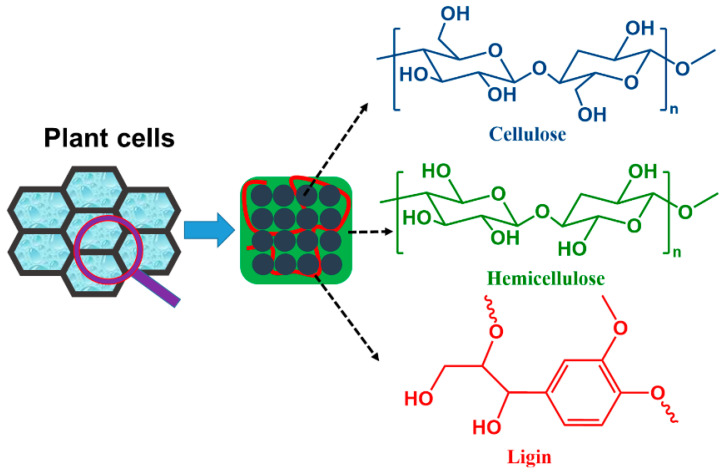
The three most common components contained in plant cell walls: cellulose, hemicellulose, and lignin, collectively known as lignocellulose.

**Figure 2:**
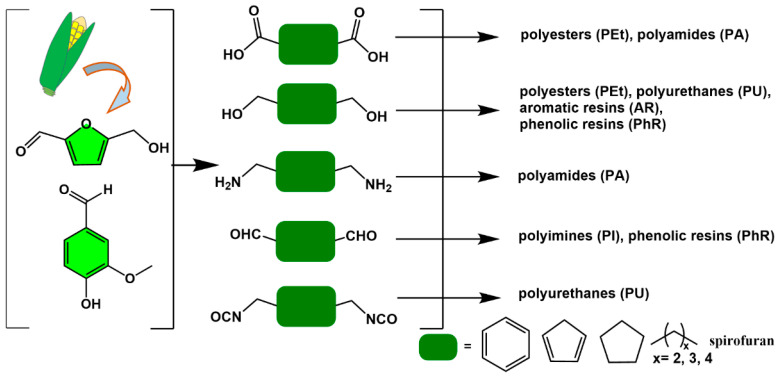
The preparation of bio-based polymers from biomass resources.

As the most abundant renewable resource on earth, lignocellulose is widely found in hardwoods (e.g., aspen), softwoods (e.g., pine needles), agricultural waste (e.g., straw), and herbaceous plants [6,7,8,9]. Its main components are cellulose, hemicellulose, and lignin (Figure 1) [10,11,12,13,14]. Depending on the type of biomass, the content of the individual components varies [4]. In wood raw materials such as hardwoods and softwoods, the cellulose content is high, up to more than 50%, while in agricultural waste such as straw, the cellulose content varies from 28% to 40%. Due to the importance of wood raw materials in structural materials and in order to reduce the diversion of food resources from biomass development, the conversion of non-edible agricultural waste into high-value-added chemicals is one of the most effective tools that have been investigated in recent years. The production of new lignocellulose-based polymers includes five steps: (1) fractionation of lignocellulose into cellulose, hemicellulose, and lignin; (2) depolymerization of the fractionated cellulose, hemicellulose, and lignin into carbohydrates and aromatic compounds; (3) catalytic or thermal conversion of the depolymerized carbohydrates and aromatic compounds to platform chemicals; (4) further conversion of the platform chemicals to the desired bio-based monomers; (5) polymerization of the above monomers to bio-based polymers by suitable polymerization methods [15,16]. This review article will focus on the progress of bio-based monomers derived from lignocellulose, in particular the preparation of bio-based monomers from 5-hydroxymethylfurfural (5-HMF) and vanillin, and their polymerization methods (Figure 2). The latest research progress and application scenarios of related bio-based polymeric materials will be also discussed, as well as future trends in bio-based polymers.

## 2. Bio-Based Monomers from Lignocellulose

### 2.1. C5/C6 Sugar Platform Chemicals Based on Cellulose/Hemicellulose

**Figure 3:**
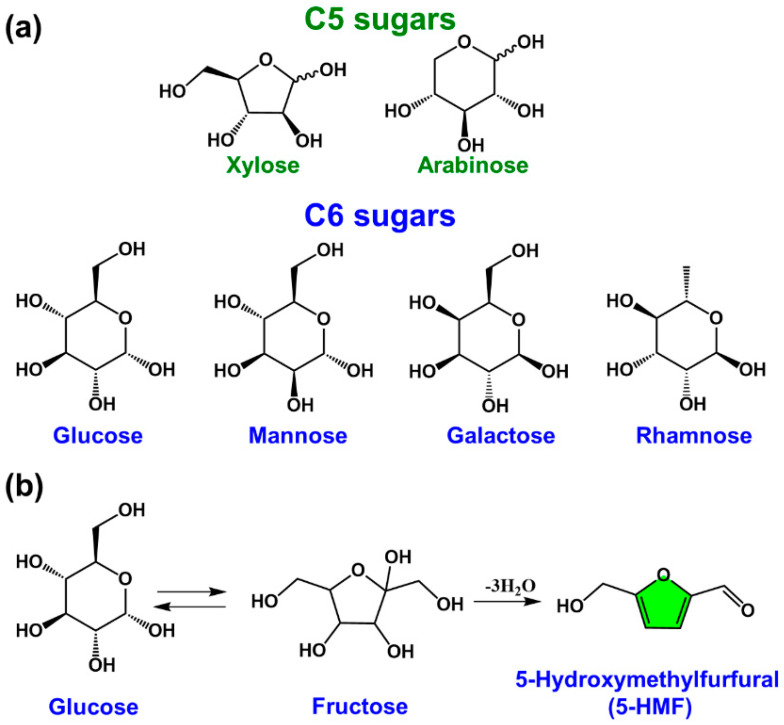
(**a**) Chemical structures of C5/C6 sugars, and (**b**) a general reaction pathway for the preparation of 5−hydroxymethylfurfural by the dehydration reaction of glucose.

Cellulose is a homopolymer of glucose and is mainly found in the cell walls of plants. The hydrolysis products of cellulose are mainly the six-membered sugar glucose (C6 sugars), and their chemical structures are shown in Figure 3a. The hydrolysis products of hemicellulose include not only glucose but also other five-membered sugars (C5: xylose, arabinose) and six-membered sugars (C6: mannose, galactose, rhamnose). These sugar intermediates can be used to prepare various first-generation furan derivatives, with furfural and 5-hydroxymethylfurfural (5-HMF) being the most common platform chemicals used to prepare polymerizable monomers (Figure 3b).

Most of the bio-based monomers are prepared based on 5-HMF, furfural, and furfuryl alcohol (FA). Of these, 5-HMF is one of the twelve core chemicals selected by the U.S. Department of Energy Biomass Program (2004) and is the most versatile intermediate. 5-HMF can be prepared highly selectively from hexoses such as fructose, and all the six carbon atoms initially in the C6 sugars are retained in the molecular structure of 5-HMF. In 2009, Binder et al. reported that a solvent of *N*, *N*-dimethylacetamide (DMAc) containing lithium chloride (LiCl)) (DMAc/LiCl) can be used to efficiently prepare 5-HMF in a single-step reaction at low temperatures (≤140 °C) [17]. Reaction mechanism studies have shown that the loosely bound halide ions in the DMAc-LiCl ion pair play a crucial role in the reaction process. In addition, Dumesic et al. studied the impact of solvent choice on HMF yield by adding inorganic salts (e.g., NaCl) to a concentrated fructose aqueous solution (30 wt%) in a biphasic system containing an organic phase. The 5-HMF yield could be increased by increasing the partitioning of 5-HMF in the extraction phase [18]. The results indicated when tetrahydrofuran (THF) was used as the organic extraction phase, high selectivity (83%) and high extracting power (R = 7.1) at 150 °C were achieved. A research team from Ehime University developed a high-temperature, high-pressure continuous flow microwave organic reactor that can achieve a special reaction operation: rapid rising of reaction temperature from room temperature to 400 °C within 0.01 s and rapid reduction of temperature after the reaction. The process can effectively suppress the generation of side reactions and increase the yield of 5-HMF to 70%. The obtained 5-HMF as a platform chemical can be converted into a variety of derivatives, which are important raw materials for bio-based polymers [19,20,21,22,23,24]. The reported types of derivatives can be divided into two categories according to whether the furan ring structure remains intact in the derivatives: (1) 5-HMF-derived monomers without furan ring structure and (2) 5-HMF-derived monomers with a furan ring structure.

### 2.2. Bio-Based Monomers without Furan Ring Structures Derived from 5-HMF

**Figure 4:**
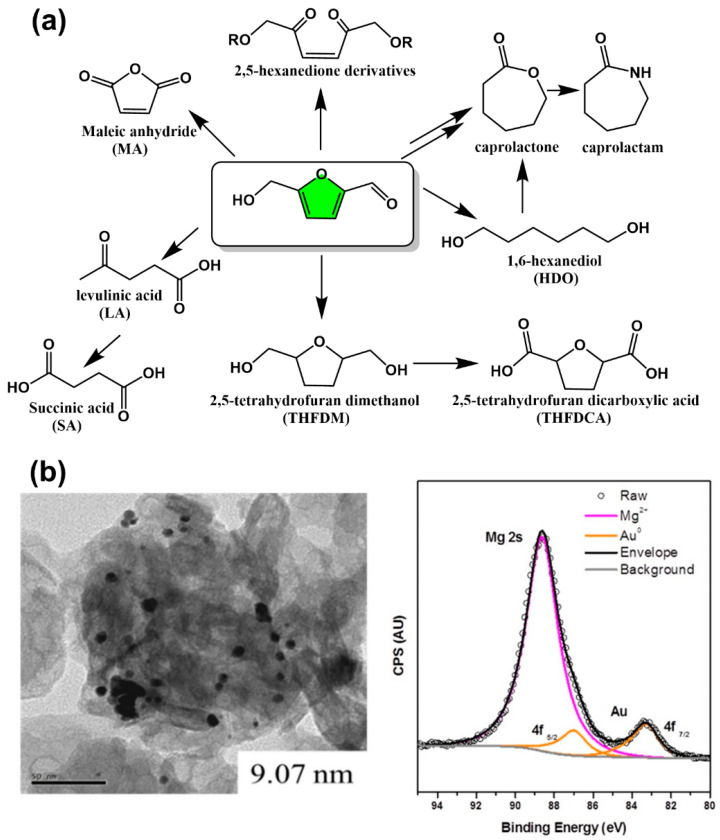
(**a**) 5-HMF-derived bio-based monomers without furan ring structures and (**b**) AuNP/HT catalyst for efficient conversion of THFDM to THFDCA [33]. Reprinted/adapted with permission from Ref. [33]. Copyright 2019, American Chemical Society.

The furan ring in the 5-HMF structure can undergo various reactions such as with electrophilic reagents, nucleophilic reagents, oxidizing agents, reducing agents, cycloadditions, and metals and metal derivatives to synthesize various bio-based monomers as well as precursors (Figure 4a) [25]. Production of 2,5-hexanedione from 5-HMF was achieved us high temperature water (HTW) as a reaction medium and Zn as a catalyst [26]. The HTW at 250 °C was desirable for acid/base catalyzed HMF conversion. Maleic anhydride is a versatile chemical intermediate used in the production of various compounds and polymers, such as 1,4-butanediol, fumaric acid, tetrahydrofuran, and polyester resins. It was abandoned to use benzene as starting chemicals for synthesis of maleic anhydride in the industry because of the toxicity and undesirable loss of two carbons for the structure during reactions. The replacement of a benzene-based process with a biomass-based process is promising. Xu et al. obtained a from-5-HMF-to-maleic anhydride yield of 52% using VO(acac)_2_ as a catalyst after a reaction at 90 °C for 4 h in acetonitrile through the simultaneous oxidation of hydroxymethyl group and C–C bond cleavage of 5-HMF [27]. Levulinic acid is an important biomass-derived platform chemical as an intermediate for the preparation of polymer monomers such as succinic acid [28,29,30]. The acid-catalyzed decomposition of 5-HMF for the preparation of levulinic acid has been widely reported, but most of them were limited by low yields and deficient economic efficiency. Recently, a new strategy for the efficient green preparation of levulinic acid using bifunctional Brønsted Lewis acids (HScCl_4_) as catalysts has been proposed by Yu et al. [31]. In addition, the catalytic hydrogenation of 5-HMF using Pd/Al_2_O_3_ catalysts afforded 2,5-tetrahydrofuran dimethanol (THFDM) with a 9:1 ratio of cis to trans structure [32]. The oxidation of THFDM in water using a hydrotalcite-supported gold nanoparticle (AuNP) catalyst (~2 wt%) gave rise to 2,5-tetrahydrofuran dicarboxylic acid (THFDCA) with a high yield of 91%, which is a compound with potential applications in the biopolymer industry (Figure 4b) [33]. The AuNP size and basicity of hydrotalcite (HT) significantly impact the performance of the catalyst and that sintering of AuNPs was the main pathway for catalyst deactivation. 1,6-Hexanediol (HDO) is a highly valuable and important compound with two hydroxyl groups at the end of the molecule. This structure makes it an ideal monomer for the synthesis of polymers such as polyesters, polyurethanes, adhesives, and unsaturated polyesters. Bio-based HDO can also be prepared by directly catalytic conversion of 5-HMF using a Pd/ZrP catalyst and formic acid as a hydrogen source in 43% yield after 21 h of reaction at 413 K [34]. HDO was also prepared by direct catalytic conversion of 5-HMF in a fixed-bed reactor using a Pd/SiO_2_ + Ir-ReOx/SiO_2_ bilayer catalyst. Under optimal reaction conditions (373 K, 7.0 MPa H_2_, mixed solvent of water (40%), and tetrahydrofuran (60%)), the yield of HDO was 57.8% [35]. The direct conversion of 5-HMF to caprolactone is difficult and therefore an indirect method is used. HDO is one of the most important intermediates and can also be prepared by oxidative cyclization of caprolactone [36]. The conversion of caprolactone to caprolactam through reacting with ammonia is a commonly used industrial process.

### 2.3. Bio-Based Monomers with Furan Ring Structures Derived from 5-HMF

**Figure 5:**
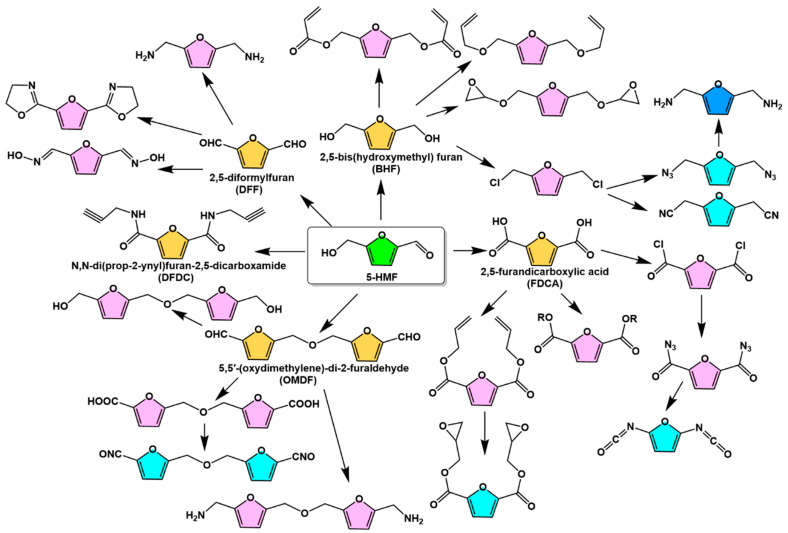
5-HMF-derived bio-based monomers containing furan ring structures [25]. Reprinted/adapted with permission from Ref. [25]. Copyright 2017, John Wiley and Sons.

The chemical modification of the hydroxyl and aldehyde groups in the 5-HMF molecular structure can also be selectively reacted to prepare bio-based monomers with the furan ring structure remaining unchanged. The monomers can be used for the synthesis of polyamides, polyesters, polyurethanes, polycarbonates, polyimides, and polyureas. Figure 5 illustrates various monomers obtained from the 5-HMF side group reactions [25]. 2,5-Furandicarboxylic acid (FDCA), 2,5-diformylfuran (DFF), and 2,5-bis(hydroxymethyl)furan (BHF) are the most common 5-HMF derived monomers with symmetrical chemical structures. These chemicals can also be used as intermediates to design the synthesis of bio-based monomers with reactive groups such as vinyl, acetylene, amine, and epoxy groups. FDCA is one of the most important near-market platform chemicals with an estimated value of USD 50.5 billion and is considered a promising alternative to petroleum-based terephthalic acid (TPA) for the production of green polymers such as polyethylene 2,5-furandicarboxylate (PEF) [37,38,39].

The production of FDCA from the oxidation of bio-based 5-HMF is of great interest and can be achieved by processes of aerobic catalytic oxidation, electrochemical catalytic oxidation, and biocatalytic reaction [40]. The most commonly used catalysts in the aerobic catalytic oxidation method are precious metal oxides (e.g., palladium catalysts: Pd-Bi-Te/C [41], platinum catalysts: CeCP@Pt [42], and gold nanoparticles loaded on metal oxides: Au/TiO_2_ [43], Au/CeO_2_ [44], Au-Cu/TiO_2_ [45], and Au/Ce_1−*x*_ Bi_*x*_ O_2−*δ*_ [46]). However, precious metal catalysts suffer from high cost, harsh reaction conditions (high reaction temperature as well as high reaction pressure), lack of universality, and non-recyclability, thus hindering large-scale industrial applications. Recently, Li et al. have reported a mild solvent-free and alkali-free reaction system with a PdO/AlPO_4_-5 catalyst under an oxygen atmosphere. [47] Using this reaction system, FDCA selectivity of up to 83.6% can be obtained at 80 °C for 5 h. Mechanistic studies showed that the hydroxyl group in the chemical structure of 5-HMF was oxidized first in this reaction system, while in the reaction that happened in the aqueous phase, the aldehyde group was oxidized first. Transition metal oxides are potential alternatives to noble metal catalysts. For instance, Hu et al. achieved near 100% conversion efficiency of 5-HMF to FDCA and up to 99.5% FDCA yield using two-dimensional Mn_2_O_3_ nanoflakes as catalysts [48].

The electrochemical oxidation of 5-HMF for the preparation of FDCA has a number of advantages, including (1) ambient reaction temperature and reaction pressure, (2) no need for high oxygen pressure, (3) no need for toxic oxidants, and (4) non-precious metals catalysts. However, the low surface area of typical catalysts results in low overall electrocatalytic activity, and the development of new catalysts with electrically active centers and high porosity is an effective way to improve reaction efficiency [49]. To address this issue, Pila et al. loaded a thin layer of Co(OH)_2_ on the surface of the porous metal–organic framework (MOF) material ZIF-67 and achieved excellent catalytic performance with conversions of up to 90.9%, FDCA yields of 81.8% and Faraday efficiencies of 83.6% [49]. Importantly, the applied potential for the reaction was reduced to 1.42 V (vs RHE), one of the lowest potentials reported.

The biocatalytic process is another synthetic method that allows the production of FDCA under mild conditions. 5-HMF oxidase and aryl alcohol oxidase can catalyze the complete oxidation of 5-HMF to FDCA [50,51]. Although a biocatalytic process generally needs a long reaction time, selecting suitable oxidases and optimizing experimental conditions could improve the reaction rate and achieve high conversion with a short reaction time. A tandem enzymatic one-pot reaction using galactose oxidase M3-5 and aldehyde oxidase PaoABC can also convert 5-HMF to pure FDCA with an isolated yield of 74% [52]. In particular, at 10 mM 5-HMF concentration, almost complete conversion (97%) of 5-HMF to FDCA after 1 h was observed. The galactose oxidase (GOase) variant prepared by W. Birmingham et al. was very active against 5-HMF, with high oxygen adsorption and very high production capacity [53]. The selective oxidation of 5-HMF to 2,5-diformylfuran was also possible. The biocatalyst and reaction conditions provide a blueprint for the further development of effective enzymatic catalysts and also facilitate the development of large-scale biocatalytic techniques for the preparation of furan-based compounds from sustainable feedstocks.

Similar to FDCA, DFF is one of the main products of the oxidative transformation of 5-HMF and is used as a chemical intermediate as a starting material for the synthesis of ligands, drugs, pesticide antifungals, fluorescent materials and new polymeric materials [54,55,56]. The oxidation of 5-HMF to DFF is always accompanied by a number of side reactions, such as the over-oxidation of DFF to FDCA, the oxidation of aldehydes to 5-hydroxymethyl-2-furancarboxylic acid (HMFCA), decarbonylation, and cross-polymerization to produce unwanted by-products. Therefore, selective oxidation of 5-HMF to DFF remains challengeable [56]. A number of catalysts have been reported for the selective oxidation of 5-HMF to DFF, such as manganese oxide-based catalysts [57], graphitized carbonitrides (g-C_3_N_4_) based catalysts [58], Ru-loaded catalysts [59], and vanadium-based catalysts [60], etc. Recently, a visible light catalyst developed by Huang et al. has achieved 95% DFF selectivity and good cycling stability [58]. The in situ oxidation using Co_3_O_4_ electrochemical catalyst developed by Zhang et al. achieved 100% conversion of 5-HMF to FDCA in 93.2% yield due to its unique defect structure and abundant electroactive sites [61]. More notably, the above reaction process simultaneously produces hydrogen, thus allowing the preparation of clean hydrogen energy as well as obtaining high-value chemicals, which can contribute to the realization of carbon neutrality from many aspects.

In addition, the catalytic hydrogenation of 5-HMF to prepare BHF is also a very important reaction for the high-value conversion of biomass. BHF can be used as a feedstock for renewable polymers such as bio-based polyesters and polycarbonates. Common catalysts are metal oxide-loaded platinum catalysts (MO/Pt), which vary greatly in their reaction selectivity depending on the type of metal oxide. Basic metal oxides such as MgO/Pt can achieve up to 99% BHF selectivity, while acidic loaded oxides such as TiO_2_/Pt have a lower BHF selectivity [62].

### 2.4. Lignin-Based Platform Chemicals and Polymeric Monomers

**Figure 6:**
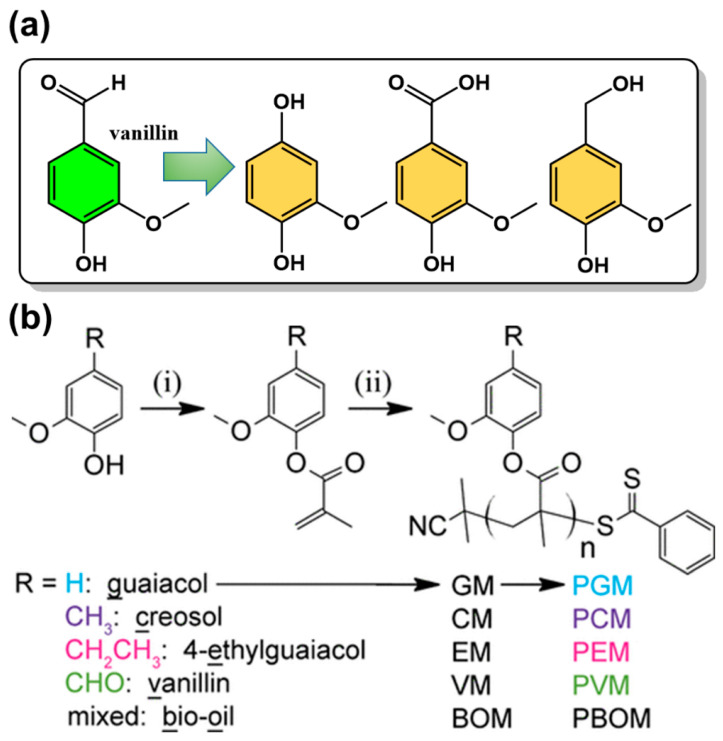
(**a**) Representative platform compounds derived from vanillin and (**b**) synthetic route of vanillin-based methacrylate monomers and the corresponding polymers. Reprinted/adapted with permission from Ref. [71]. Copyright 2016, American Chemical Society.

The aromatic structure of lignin dictates that its degradation products are platform compounds with unique methoxyphenol structures [63]. However, due to its structural complexity and heterogeneity, its selective degradation of lignin to obtain chemicals with high selectivity and yield is very challenging. The main approaches to make lignin depolymerization include thermochemical (e.g., hydrolysis, gasification, hydrothermal liquefaction, and microwave methods), chemical (e.g., acid-base catalysis, ionic liquids/supercritical fluids, oxidation,) and biotechnological (e.g., bacterial, fungal, enzymatic) routes [64,65]. The resulting platform chemicals can be structurally simpler compounds by the selective breaking of C-O bonds; new fine chemicals and bio-based monomers can then be prepared by selective modification of specific functional groups [66,67,68,69]. 3-Methoxy-4-hydroxybenzaldehyde (vanillin) is one of the most important platform compounds derived from lignin conversion, containing both aldehyde and hydroxyl groups for further functionalization. It can be used as an intermediate compound for the preparation of a variety of bio-based monomers with aromatic structures (Figure 6a). Vanillin is the only commercially available bio-based aromatic compound because the depolymerization and purification of lignin are complex and difficult. Therefore, it has received a great deal of attention from the polymer community and can be used as a starting material for the preparation of various vanillin-based polymers, such as phenolic resins, benzoxazine resins, polyesters, acrylates, and methacrylate polymers (Figure 6b) [70,71]. A derivative, vanillin methacrylate was also reported as a bio-based monomer for the synthesis of aldehyde-containing porous materials [72]. The hydroxyl group in vanillin can also react with epichlorohydrin to synthesize compounds with both epoxy and aldehyde groups for the preparation of epoxy resins.

## 3. Common Polymerization Methods of Bio-Based Monomers

Since most polymers are synthesized depending on the petrochemical industry, which always accompanies a negative impact on the environment, there is a strong need to develop a facile and efficient synthesis of bio-based polymers using renewable platform chemicals as monomers [73]. Similar to petroleum-based monomers, the polymerization of bio-based monomers can be divided into chain-growth and step-growth polymerization depending on the reaction mechanism proposed by Flory in 1953 [74]. In chain-growth polymerization, the reaction starts from the active center generated by initiators and consists of chain initiation, chain propagation, and chain termination. The reaction rates and activation energies vary considerably between the various stages. Common free radical polymerization, ionic polymerization, coordination polymerization, living polymerization, and ring-opening polymerization belong to the category of chain-growth polymerization. Different from chain-growth polymerization which is proceeded by the addition of a monomer to a growing polymer with an active center, step-growth polymerization is gradually proceeded by reactions between any two compounds of monomers and growth chains with the rate and activation energy of each reaction step being approximately the same. Polymers including polyesters, polyamides, polyurethanes, etc., are produced through step-growth polymerization. Since most bio-based monomers contain functional groups such as hydroxyl, amine, and carboxyl groups, step-growth polymerization is the most common method for the preparation of bio-based synthetic polymers. Ring-opening polymerization is also used for the polymerization of cyclic molecules such as caprolactones. Although it is rare, there are some bio-based monomers with C=C double bonds by chemically modifying 5-HMF possible for free radical polymerization.

### 3.1. Step-Growth Polymerization by Condensation or Addition

**Figure 7:**
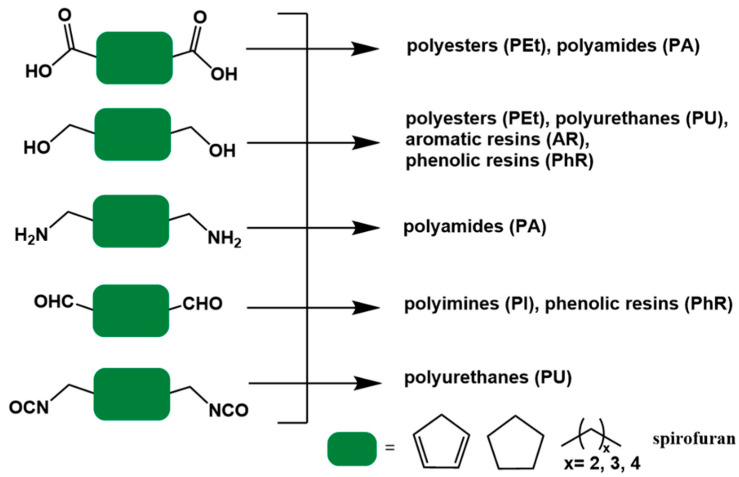
5-HMF-derived bio-based monomers for polymer species. (The green block is used for structure simplification).

In step-growth polymerization, condensation polymerization, and addition polymerization are referring to the formation of a polymer from bifunctional monomers with the release of small molecules or not. In the former, the molecular weight of the repeating units in the polymer is less than that of the monomer, e.g., the dehydration condensation polymerization of a dicarboxylate monomer with a diol. In the latter, the molecular weight of the repeating units in the polymer is same as that of the monomer, such as the reaction of diisocyanate with a diol to form a polyurethane. Figure 7 illustrates the types of polymers that can be prepared from 5-HMF derived bio-monomers, including polyesters (PEt), polyamides (PA), aromatic resins (AR), phenolic resins (PhR), polyimides (PI), and polyurethanes (PU).

Among all 5-HMF derived bio-monomers, FDCA is the most promising alternative to the petroleum-based monomers, sharing the same carboxyl functional groups and similar aromatic center and reactivity with terephthalic acid (PTA), allowing the preparation of high-performance bio-based polymeric materials by condensation polymerization with diols or diamines [75,76,77,78,79]. However, before 2009, the development of FDCA-based polymers was limited by the purity and the price of the bio-monomer feedstocks. With the rapid development of biotechnology and the chemical industry, the economics and purity of FDCA feedstock have been greatly improved. In 2009, Gandini et al. prepared poly(ethylene glycol) 2,5-furandicarboxylate (PEF) by performing a transesterification and polycondensation between FDCA dimethyl ester with ethylene glycol using an antimony oxide catalyst (Sb_2_O_3_, 70–200 °C under high vacuum) and obtained high-molecular-weight products (degree of polymerization: 250–300) [76]. Since then, different diols such as 1,3-propanediol, 1,4-butanediol, 1,6-hexanediol, and 1,8-octanediol have been used as monomers in condensation reactions with FDCA to prepare bio-based polyesters containing different alkyl chain lengths [77].

FDCA-derived bio-based polyesters are non-toxic, renewable, and have controlled degradation and similar properties to polymers of PTA and diols (e.g., polyethylene glycol terephthalate, PET) in terms of glass transition temperature (*T_g_*), melting point (*T_m_*), Young’s modulus (*η*), and tensile strength (*σ*). Table 1 compares the thermomechanical properties of PEF and PET as well as polybutylene glycol 2,5-furandicarboxylate (PBF) and polybutylene glycol terephthalate (PBT) [77,78,79]. Although FDCA and TPA have very similar structures, there are differences in aromatic ring size, molecular polarity, and molecular symmetry, e.g., the distance between the two carboxyl groups in TPA is 5.731 Å, whereas in FDCA this value is less than 4.830 Å [78]. In addition, the angle between the two carboxylic acid groups is different, 180° in TPA and 129.4° in FDCA. These structural differences therefore lead to some specific properties of PEF, such as better gas barrier properties than PET, because of the polarity of the furan ring which could favorably interact with polar molecules. In particular, PEF has 6 times higher O_2_ barrier than PET and 14 times higher CO_2_ barrier than PET, thus offering great potential for applications such as high-performance food packaging materials. PET and PEF have similar chemical structures, which makes their recycling face similar problems. For instance, under a mechanical recycling process, incomplete separation and recovery of pure streams are general problems limited by technical difficulties. The degradation properties of the PEF in comparison with the PET were also well studied [80]. It was found that the PEF films degraded 1.7 times faster than PET ones under enzymatic hydrolysis. For instance, not only in the academic field, with the growth of global environmental issues and for achieving carbon neutrality by 2050, various large chemical companies such as Coca-Cola, DuPont, BASF, Mitsubishi, and Avantium are also vigorously pursuing the application of bio-based polymers instead of traditional petroleum-based materials. They are devoted to the commercialization of PEF and its copolymers. Among them, Avantium’s 100% bio-based PEF, developed based on YXY technology, has been used in renewable plastic bottles, fibers as well as films, and other commodities [4].

### 3.2. Chain-Growth Polymerization

#### 3.2.1. Ring-Opening Polymerization

Ring-opening polymerization (ROP) of cyclic bio-based monomers is a common method for the production of bio-based polymers, for instance, ROP of bio-based caprolactones [81]. The ROP products of caprolactones are aliphatic polyesters with biodegradability, good compatibility with other polymers, and easy processing. As U.S. Food and Drug Administration (FDA)-approved materials for human use, bio-based polycaprolactones (PCLs) have a wide range of applications in resin modification, coatings, drug-carrying materials, binders, etc. [82]. Depending on the type of initiator used, the ROP mechanism varies by anionic ROP, cationic ROP, and acid-activated monomer ROP mechanisms [83].

**Figure 8:**
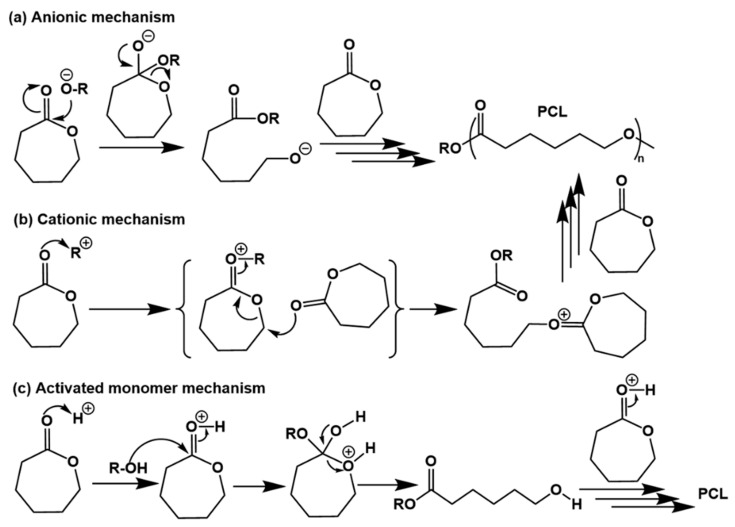
Reaction mechanism for ring-opening polymerization of caprolactones: (**a**) anionic polymerization mechanism, (**b**) cationic polymerization mechanism, (**c**) acid-activated monomer mechanism.

In the anionic ROP, nucleophilic reagents such as organometallics, alcohol salts and alcohols are used as initiators. The mechanism of polymerization is shown in Figure 8a, where an initiator such as an alcohol salt attacks the carbonyl carbon in the lactone monomer to open the ring structure of the lactone and then creates growing anionic chain ends. It is worth noting that the reaction produces a number of side reactions such as inter- and intra-molecular ester exchange reactions leading to the shortening of the polymer chain or lengthening of the chain, thereby expanding the molecular weight distribution. In addition, some reverse tail-bite reactions can also occur leading to the formation of oligomeric macrocyclic molecules. In contrast to the anionic mechanism, the cationic initiator in the cationic ROP first electrophilically attacks the carbonyl oxygen atom to produce an oxonium ion, which is a class of cation with an oxygen substituent attached to the central sp_2_ hybridized carbon atom and capable of carrying a positive charge through π-bond dispersion between the central carbon and oxygen atoms. This then leads to alkyl oxygen cleavage and growth occurs through the continued reaction of this oxonium ion with the monomers (Figure 8b). Because the growing polymer chain end carries a positively charged terminus, the cationic mechanism is also known as the active chain end mechanism. Unlike the cationic mechanism where the active chain end is growing, a proton ion is added to the monomer side to continuously generate activated monomers for chain growth in the activated monomer mechanism (Figure 8c). First, the protonated lactone cation is approached by the hydroxyl nucleophilic reagent at the chain end, which opens the C=O double bond and leads to ring opening. The ring-opening reaction via the activation monomer mechanism is less susceptible to side reactions or molecular rearrangements because it does not have charged chain ends.

#### 3.2.2. Free Radical Polymerization

**Figure 9:**
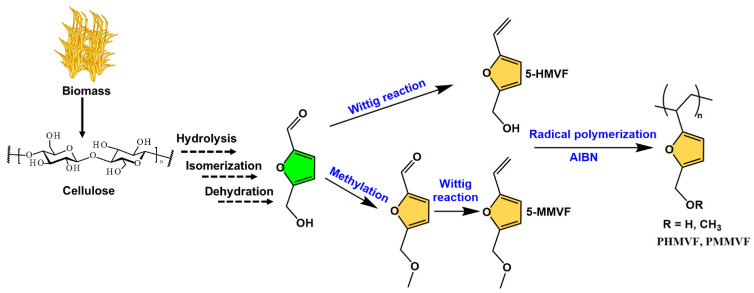
Synthetic routes of 5-HMVF and 5-MMVF bio-based monomers and their free radical polymerization.

Free radical polymerization is carried out in the mechanism of chain-growth polymerization initialized by an initiator. The application of 5-HMF as a direct feedstock in free radical polymerization is less common. However, the aldehyde group in 5-HMF can be selectively modified to other chemical structures suitable for free radical polymers, such as vinyl, acetylene-based monomers, etc. In 2008, Yoshida et al. transformed 5-HMF into 2-hydroxymethyl-5-vinylfuran (5-HMVF) and 2-methoxymethyl-5-vinylfuran (5-MMVF) by the Wittig reaction (Figure 9) [84]. 5-MMVF requires a methylation step to convert the hydroxyl group to methoxy group. Polymerization of these monomers is achieved at 70 °C under a nitrogen atmosphere using azodiisobutyronitrile (AIBN) as an initiator. The resulting polymers, PHMVF and PMMVF are in the form of oligomers and have number average molecular weights (M_n_) of 2170 and 2890 g/mol, respectively.

## 4. Conclusions and Future Prospects of Bio-Based Polymers

For any new material, moving from concept to market launch is a challenge, with many hurdles to overcome, including the technology required to scale up the production and processing of the material. In recent years, the development of bio-based monomers and polymers as well as qualitative improvements in mechanistic research and commercialization have advanced by leaps and bounds, for example, the commercialization of PEF. However, the market share of bio-based polymers is still only about 1%, so there is still a long way to go in terms of their popularity and dissemination. This further requires increased research and development efforts in the areas of large-scale and efficient preparation of bio-based monomers, the achievement of improved properties of the bio-based polymers to approach those of petroleum-based polymers, and new synthetic technologies. In the production of bio-based monomers, conversion studies for different lignocellulosic feedstocks, the development of efficient production units, and the design of new catalytic systems may help to achieve more efficient and economical production of bio-based monomers than petroleum-based monomers [85].

**Figure 10:**
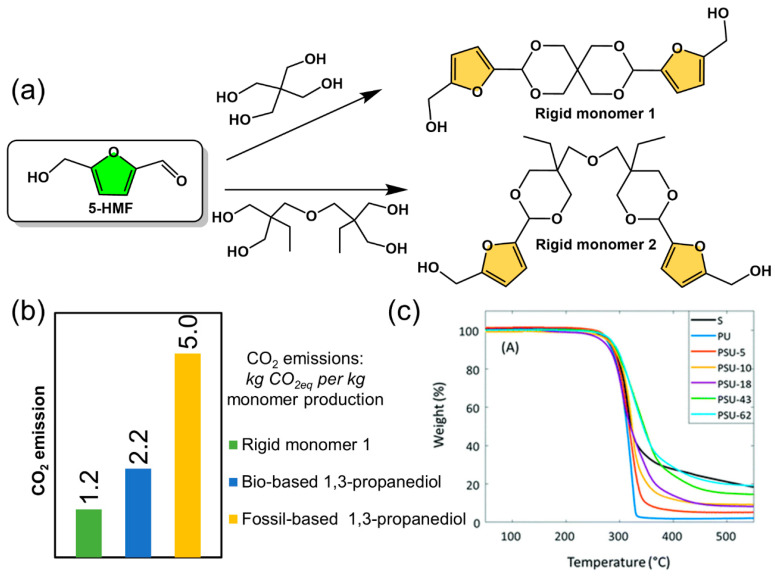
(**a**) Novel bio-based rigid monomers derived from 5-HMF in spirofuranyl, (**b**) CO_2_ emission during monomer production and (**c**) TGA curved of rigid monomer 1 (black line) and its poly(urea-urethane) copolymers. Reprinted/adapted with permission from Ref. [86]. Copyright year, Royal Society of Chemistry.

In terms of polymer performance, bio-based polymers that have been commercialized or are close to commercialization are still lacking in terms of high performance and therefore require further optimization of molecular design and synthetic process. For petroleum-based polymer materials, hard monomers with aromatic structures characterized by high glass transition temperature (*T_g_*) can provide the polymer with higher mechanical strength and heat resistance temperatures. In recent years, the design and synthesis of bio-based hard monomers have been exploited to develop high performance polymeric materials for replacement of engineering plastics. Some bio-based hard monomers were used to obtain polymers with high glass transition temperatures, such as spirofuran-based monomers (Figure 10) [86,87,88,89,90,91,92,93]. It is also found that the CO_2_ emission for production of the bio-based rigid monomer 1 (Figure 10a,b) was much lower than that of fossil-based monomers, which gave rise to high performance polymers with a degradation temperature of up to 300 °C based on thermal gravimetric analysis (TGA) (Figure 10c) [86]. In addition to this, advanced computational methods can be used in the design of bio-based monomer structures to quickly and efficiently select suitable bio-based monomers among numerous molecular structures and to design rational polymerization routes, for example, material informatics methods using domain modeling to explore polymer structure–property relation [94].

## Figures and Tables

**Table 1:** Comparison of various properties of PEF vs. PET and PBF vs. PBT.

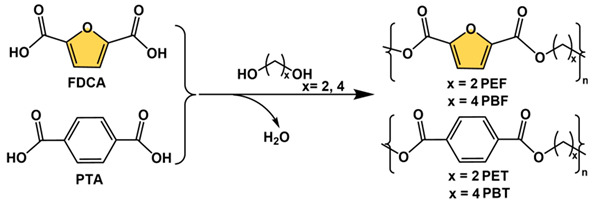						
Polymer	*T_g_*	*T_m_*	*T_d_*	*η*	*σ*	*M_n_*
	(°C)	(°C)	(°C)	(GPa)	(MPa)	(kg/mol)
PEF	82–89	210–250	389	2.5–2.8	67–85	83–105
PET	71–79	246–260	407	2.0–2.5	65–72	6.4
PBF	36–44	169–172	373	1.8–2.0	55–62	11.8–17.8
PBT	24–48	220–227	384	1.4–1.6	51–56	17.7–44

## Data Availability

No new data were created.
